# Nomogram predict relapse-free survival of patients with thymic epithelial tumors after surgery

**DOI:** 10.1186/s12885-021-08585-y

**Published:** 2021-07-22

**Authors:** Yang-Yu Huang, Lei-Lei Wu, Xuan Liu, Shen-Hua Liang, Guo-Wei Ma

**Affiliations:** 1 State Key Laboratory of Oncology in South China, Sun Yat-sen University Cancer Center, Collaborative Innovation Center for Cancer Medicine, Guangzhou, China; 2 Department of Thoracic Surgery, Shanghai Pulmonary Hospital, School of Medicine, Tongji University, Shanghai, P. R. China

**Keywords:** Thymic epithelial tumor, Overall survival, Nomogram, Albumin, Ratio of neutrophils to lymphocytes

## Abstract

**Background:**

Hematological indicators and clinical characteristics play an important role in the evaluation of the progression and prognosis of thymic epithelial tumors. Therefore, we aimed to combine these potential indicators to establish a prognostic nomogram to determine the relapse-free survival (RFS) of patients with thymic epithelial tumors undergoing thymectomy.

**Methods:**

This retrospective study was conducted on 156 patients who underwent thymectomy between May 2004 and August 2015. Cox regression analysis were performed to determine the potential indicators related to prognosis and combine these indicators to create a nomogram for visual prediction. The prognostic predictive ability of the nomogram was evaluated using the consistency index (C-index), receiver operating characteristic (ROC) curve, and risk stratification. Decision curve analysis was used to evaluate the net benefits of the model.

**Results:**

Preoperative albumin levels, neutrophil-to-lymphocyte ratio (NLR), T stage, and WHO histologic types were included in the nomogram. In the training cohort, the nomogram showed well prognostic ability (C index: 0.902). Calibration curves for the relapse-free survival (RFS) were in good agreement with the standard lines in training and validation cohorts.

**Conclusions:**

Combining clinical and hematologic factors, the nomogram performed well in predicting the prognosis and the relapse-free survival of this patient population. And it has potential to identify high-risk patients at an early stage. This is a relatively novel approach for the prediction of RFS in this patient population.

**Supplementary Information:**

The online version contains supplementary material available at 10.1186/s12885-021-08585-y.

## Background

Thymic epithelial tumors common occur in the anterior mediastinum and can be divided into thymoma and thymic carcinoma according to histology [1, 2]. The Masaoka-Koga staging system, which is based on the progression of the primary tumor and the degree of involvement of the surrounding organs, has been widely accepted for thymoma and thymic carcinoma [3–5]. However, Yanagiya et al. found that age and histological type were significant prognostic factors in their cohort, which were not observed or reported in the Masaoka-Koga staging system [6]. Similarly, the results of the study published by Fukui et al. revealed that the new classification showed a better prognostic effect for thymic tumors than the Masaoka-Koga classification [7, 8]. Moreover, compared with the staging systems for most other malignant tumors, the Masaoka-Koga system does not include the effect of lymph node or distant organ metastasis on prognosis as finely as the TNM staging.

At the same time, an increasing number of studies have used clinical factors such as history of hypertension, diabetes, [9] smoking, [10] and body mass index (BMI) [11] and hematological indicators including hemoglobin (Hb) [12], neutrophil-to-lymphocyte ratio (NLR) [13, 14], albumin (ALB) [15], and other such indicators to analyze the prognosis of various tumors. However, only few studies have comprehensively analyzed a combination of the two types of indicators to establish a prognostic model for patients with thymic epithelial tumors after thymectomy. Currently, nomograms have been developed for most cancer types [16–18]. Compared with the traditional staging system for many cancers, the use of a nomogram has advantages in terms of prognostic prediction. Therefore, it has been proposed as an alternative method for cancer staging [19–21].

Therefore, in this study, we aimed to use both preoperative hematological indicators and clinical factors to construct a prognostic predictive nomogram for patients with thymic epithelial tumors after thymectomy for a comprehensive evaluation. In addition, the nomogram score was used for risk stratification to identify high-risk patients.

## Materials and methods

### Study population

This study was approved by the Medical Ethics Committee of Sun Yat-sen University Cancer Center (SYSUCC; Approval No. B2020–353-01) and complies with the Declaration of Helsinki.

**Fig. 1:**
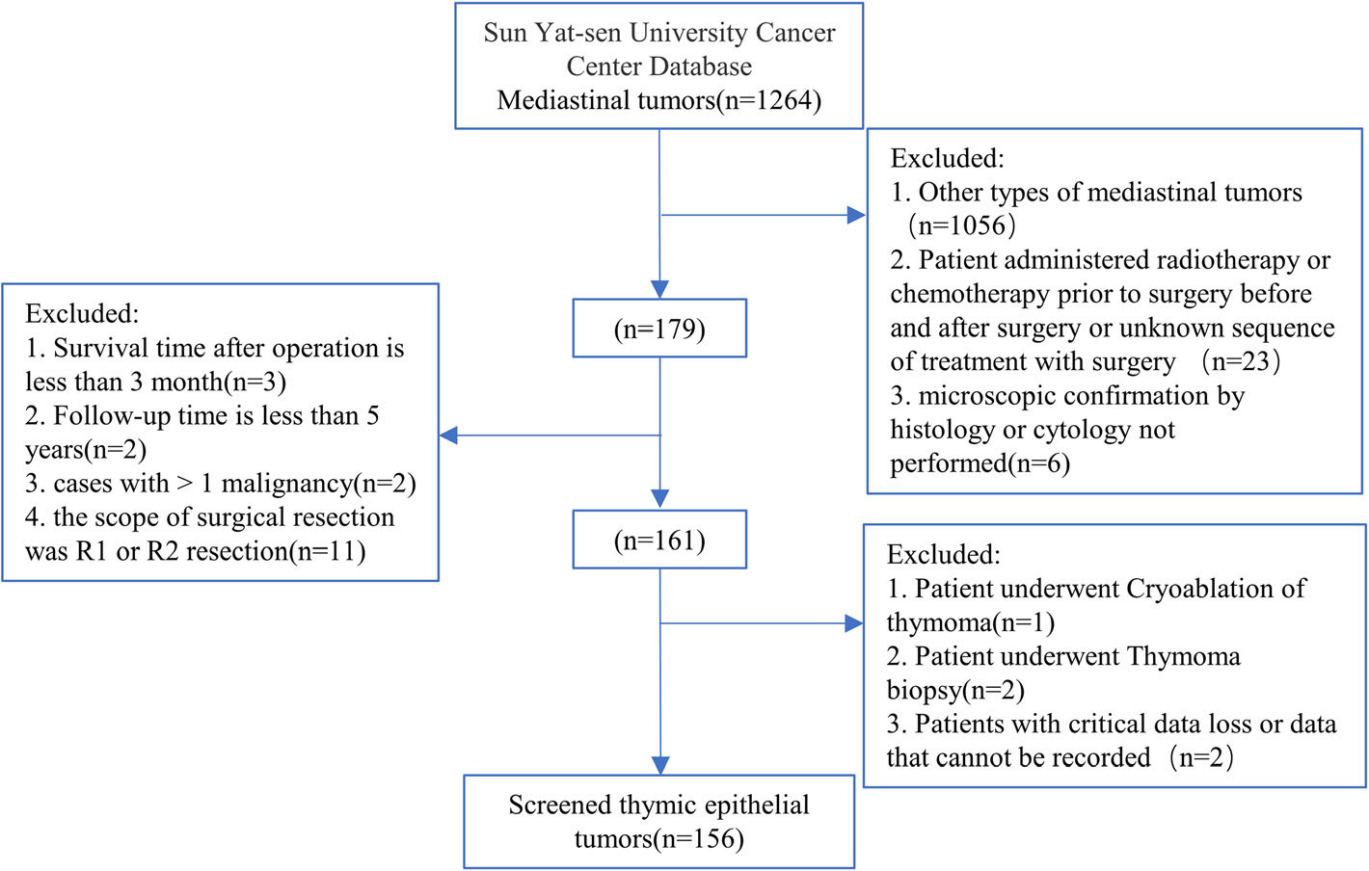
Flow chart of patient screening

This study retrospectively analyzed 156 patients who underwent R0 resection of thymus epithelial tumor at SYSUCC between May 2004 and August 2015. Most patients were included in the training group (*n* = 116), and the remaining patients were included in the verification group (*n* = 40). The inclusion criteria were as follows: (1) patients older than 18 years; (2) patients who underwent thymectomy at our center; (3) presence of histopathologically confirmed thymic epithelial tumors; (4) related laboratory examinations (blood routine, biochemical routine and so on) were completed within 7 days before the operation. 5) the scope of surgical resection was R0 resection. The exclusion criteria and the screening process are shown in Fig. 1.

### Clinical data collection

Data were collected for the following clinical variables: hematological indicators (obtained within 1 week before the operation), neutrophil count (NE), lymphocyte count (LY), platelet count, albumin levels, globulin levels as well as patients’ sex, age, smoking history, drinking history (Drinking alcohol every day, and the specific amount of drinking is not limited and described), family history of tumor, underlying disease (hypertension and/or diabetes), tumor size, histological subtype, myasthenia gravis symptoms, tumor capsule status (complete or incomplete), great vessel infiltration, Masaoka-Koga staging, and T staging. In addition, T staging and Masaoka staging were obtained by combining imaging data with intraoperative records and postoperative pathological information, we staged all patients according to the eighth edition of the TNM staging system and the modified Masaoka-Koga staging system.

### Follow-up

We followed up patients regularly. In the first 2 years, all patients were followed up every 6–12 months, every 12 months from the third to the fifth year, and then an annual follow-up was continued. The last follow-up date was August 22, 2020. The methods of follow-up were telephone and outpatient. The primary observational endpoint was RFS. RFS was defined as the length of time after primary treatment for a cancer ends that the patient survives without any signs or symptoms of that cancer.

### Statistical analysis

Statistical analysis was performed using SPSS 25.0 (IBM, Chicago, Illinois, USA) and R software (version 4.0.3; https://www.r-project.org/). In addition to age and tumor size, each component was converted to binary according to the best cutoff value (using X-tile software; http://www.tissuearray.org/rimmlab) according to the best cutoff value defined by the minimum *P* value method variables. This method has also been applied in other studies [22]. In the training cohort, the Cox regression model was used to analyze risk factors through univariate and multivariate analyses. Univariate analysis was performed to determine important risk factors for RFS. Variables with *P* values of less than 0.05 were further included in the multivariate Cox proportional hazard regression model. In the final multivariate analysis, P values of less than 0.05 were considered independent prognostic factors. Then, based on the results of the multivariate Cox analysis, we constructed a nomogram showing three-year and five-year relapse-free survival rates.

The R statistical software packages “rms,” “survival,” “foreign,” “survivalROC,” and “rmda” were used to calculate the C index; to generate the calibration curve, receiver operating characteristic (ROC) curves, decision curve analysis (DCA) curve, and Kaplan-Meier (KM) curve; and to construct a nomogram. The nomogram was used to calculate the prognostic risk score for each patient; X-tile was used to divide the patient’s score into different risk levels (low risk, medium risk, and high risk) and to show their stratification effect through the KM curve [23]. The C-index, DCA curve, and ROC curve were used to evaluate the predictive ability of the nomogram. All statistical tests were two-sided, and *P* values of less than 0.05 were considered statistically significant.

## Results

### Basic characteristics

A total of 156 patients participated in the study. Among them, 116 patients (Approximate 70%) were randomly assigned to the training group to build a nomogram, and the remaining 40 patients (Approximate 30%) were assigned to the verification group. Table 1 shows the data of the clinicopathological characteristics of the 156 patients. The three-year and five-year relapse-free survival rates were 0.932 and 0.905, respectively. We found that 129 patients (81.1%) achieved a five-year relapse-free survival time in all patients. These clinicopathological factors did not differ significantly between the training and validation cohorts.

**Table 1:** Patient, tumor, and treatment-related characteristics of thymic tumor (*n* = 156)

Characteristic	Training Cohort(*n* = 116)		Validation Cohort(*n* = 40)	
	N	%	N	%
Gender				
Male	63	54.3	20	50.0
Female	53	45.7	20	50.0
Age (years)				
≤ 60	89	76.7	32	80.0
>60	27	23.3	8	20.0
Smoking history				
Never	84	72.4	34	85.0
Ever	32	27.6	6	15.0
Drinking history				
No	100	86.2	37	92.5
Yes	16	13.8	3	7.5
Family history of tumor				
No	98	84.5	34	85.0
Yes	18	15.5	6	15.0
Underlying diseases				
No	82	70.7	34	85.0
Yes	34	29.3	6	15.0
Tumor size (cm)				
≤ 6	65	56.0	24	60.0
>6	51	44.0	16	40.0
pT stage				
T1	91	78.4	31	77.5
T2–3	25	21.6	9	22.5
Masaoka stage				
I	61	52.6	15	37.5
II-III	55	47.4	25	62.5
WHO stage				
A-AB	45	38.8	18	45.0
B1-B3	60	51.7	17	42.5
C	11	9.5	5	12.5
Myasthenia gravis,				
No	107	92.2	37	92.5
Yes	9	7.8	3	7.5
tumor capsule status				
Incomplete	38	32.8	9	22.5
Complete	78	67.2	31	77.5
Invasion of great vessels				
No	91	78.4	33	82.5
Yes	25	21.6	7	17.5
ALB				
≤ 40.8	29	25.0	7	17.5
>40.8	87	75.0	33	82.5
GLB				
≤ 28.8	58	50.0	6	15.0
>28.8	58	50.0	34	85.0
A/G				
≤ 1.5	54	46.6	18	45.0
>1.5	62	53.4	22	55.0
NE				
≤ 5.6	102	87.9	32	80.0
>5.6	14	12.1	8	20.0
LY				
≤ 2.5	28	24.1	8	20.0
>2.5	88	75.9	32	80.0
NE/LY (NLR)				
≤ 3.0	100	86.2	32	80.0
>3.0	16	13.8	8	20.0
PLT				
≤ 237	64	55.2	16	40.0
>237	52	44.8	24	60.0
PLT/LY (PLR)				
≤ 161.6	104	89.7	32	80.0
>161.6	12	10.3	8	20.0
PLT/NE*LY (SII)				
≤ 764.3	102	87.9	30	75.0
>764.3	14	12.1	10	25.0

*NLR* neutrophil-to-lymphocyte ratio, *Hb* hemoglobin, *ALB* albumin, *BMI* body mass index, *NE* neutrophil count, *LY* lymphocyte count, *GLB* globulin, *SII* systemic immune-inflammation Index, *PLT* platelet, *PLR* platelet-lymphocyte ratio, *pT stage* pathological T stage

### Univariable and multivariable analyses in the training cohort

According to the results of univariate Cox regression analysis, there were nine variables related to RFS: WHO histologic types, T stage, Tumor capsule status, Invasion of great vessels, ALB, Neutrophils (NE), NLR, PLR and systemic immune-inflammation Index (SII) (Table 2). In the multivariate Cox regression analysis, four parameters were defined as independent prognostic factors of RFS: T stage (T1 vs. T2–3, hazard ratio, HR = 7.518, 95% confidence interval, CI [1.355–41.718], ALB (HR = 0.157, 95% CI [0.035–0.697]), WHO histologic types (A-AB vs B1-B3, HR = 0.379, 95%CI [0.070–2.040], A-AB vs C, HR = 5.892, 95% CI [0.660–52.582]), and NLR (HR = 15.426, 95% CI [1.759–135.300]) (Table 2).

**Table 2:** Univariate and multivariate analysis results in Training cohort(n = 116)

Variable	Univariate analysis	Multivariate analysis		
	P	HR	95%CI	P
Gender Male vs Female	.545			
Age (years) ≤60 vs >60	.175			
Smoking history Never vs Ever	.516			
Drinking history No vs Yes	.128			
Family history of tumor No vs Yes	.858			
Underlying diseases No vs Yes	.176			
Tumor size ≤6 vs >6	.225			
pT stage	.020	Reference		
T1 vs T2–3		7.518	1.355–41.718	.021
Masaoka stage I vs II-III	.097			
WHO stage	.015	Reference		.016
A-AB vs B1-B3		.379	.070–2.040	
A-AB vs C		5.892	.660–52.582	
Myasthenia gravis, No vs Yes	.434			
tumor capsule status Incomplete vs Complete	.000			.088
Invasion of great vessels No vs Yes	.000			.117
ALB	.001	Reference		
≤40.8 vs >40.8		.157	.035–.697	.015
GLB ≤28.8 vs >28.8	.446			
A/G ≤1.5 vs >1.5	.344			
NE ≤5.6 vs >5.6	.004			.261
LY ≤2.5 vs >2.5	.305			
NE/LY (NLR)	.000	Reference		
≤3.0 vs >3.0		15.426	1.759–135.300	.014
PLT ≤237 vs >237	.075			
PLT/LY (PLR) ≤161.6 vs >161.6	.041			.082
PLT/NE*LY (SII) ≤764.3 vs >764.3	.010			.196

*NLR* neutrophil-to-lymphocyte ratio, *Hb* hemoglobin, *ALB* albumin, *BMI* body mass index, *NE* neutrophil count, *LY* lymphocyte count, *GLB* globulin, *SII* systemic immune-inflammation Index, *PLT* platelet, *PLR* platelet-lymphocyte ratio, *pT stage* pathological T stage

### Establishment of the nomogram

**Fig. 2:**
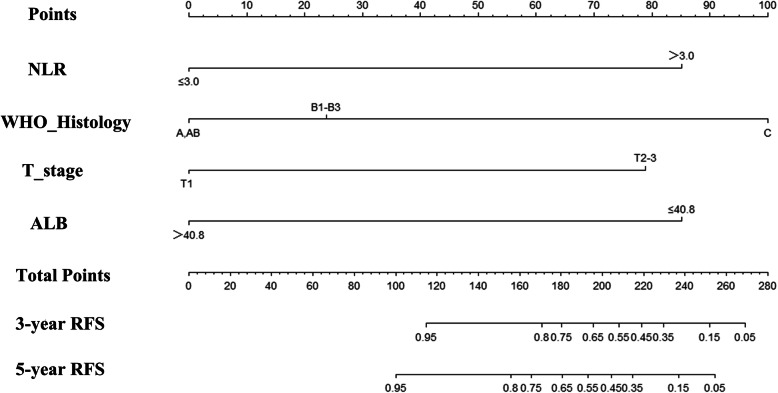
Nomogram predicting 3- and 5- relapse-free survival after thymectomy for thymic epithelial tumors patients

**Fig. 3:**
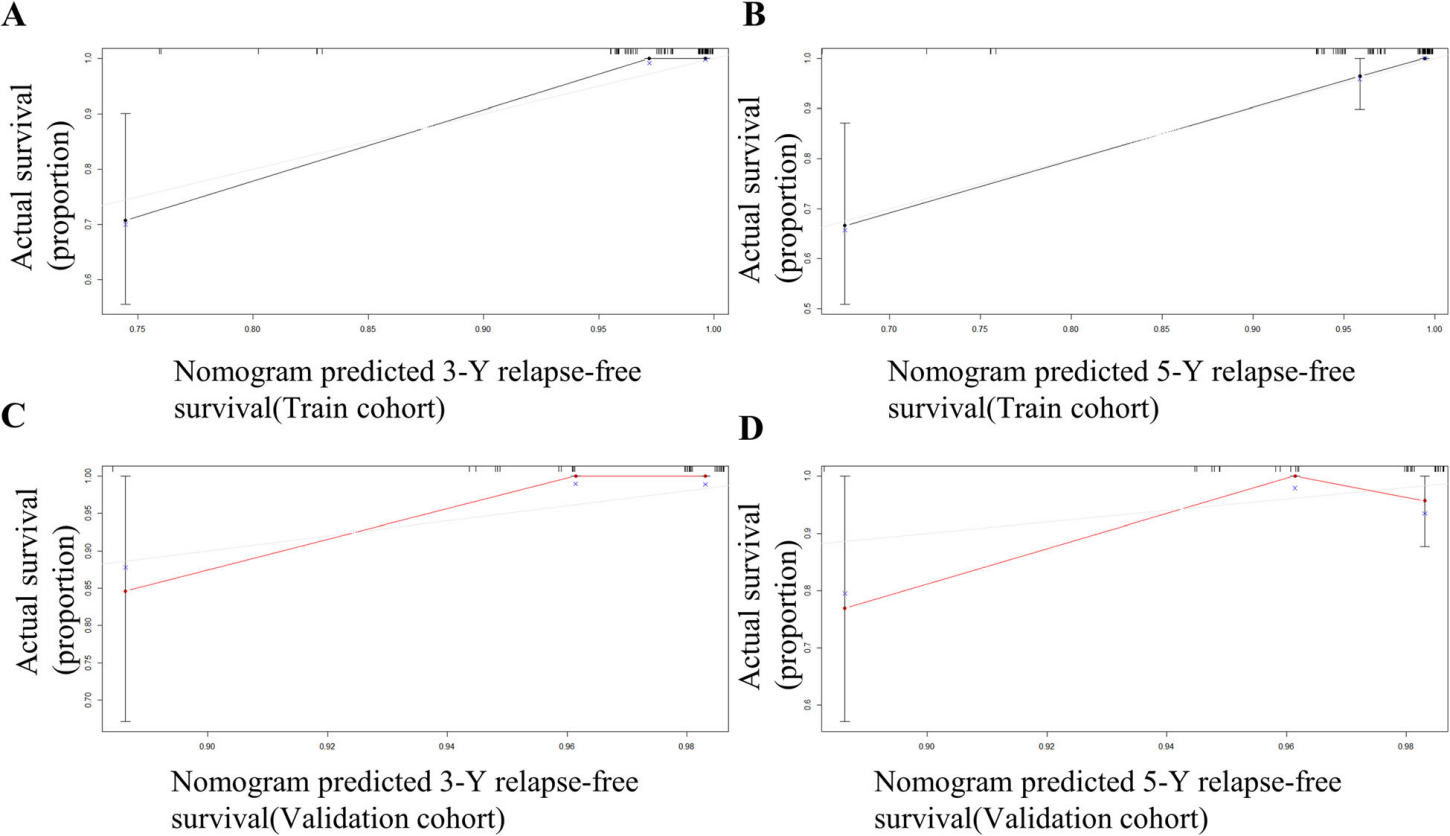
The calibration curves for predicting patient relapse-free survival at (**A**, **C**) 3-y and (**B**, **D**) 5-y in the training and validation cohorts

According to the results of the multivariate Cox regression analysis, T stage, ALB, WHO histologic types, and NLR were defined as independent prognostic factors, and these factors were integrated to form a nomogram (Fig. 2). In the training cohort, the C index was 0.902 (95% CI: 0.843–0.961). Internal calibration curves for the three- and five-year RFS closely matched those of the baseline in the training cohort (Fig. 3Aand B).

### Verification of the nomogram

**Fig. 4:**
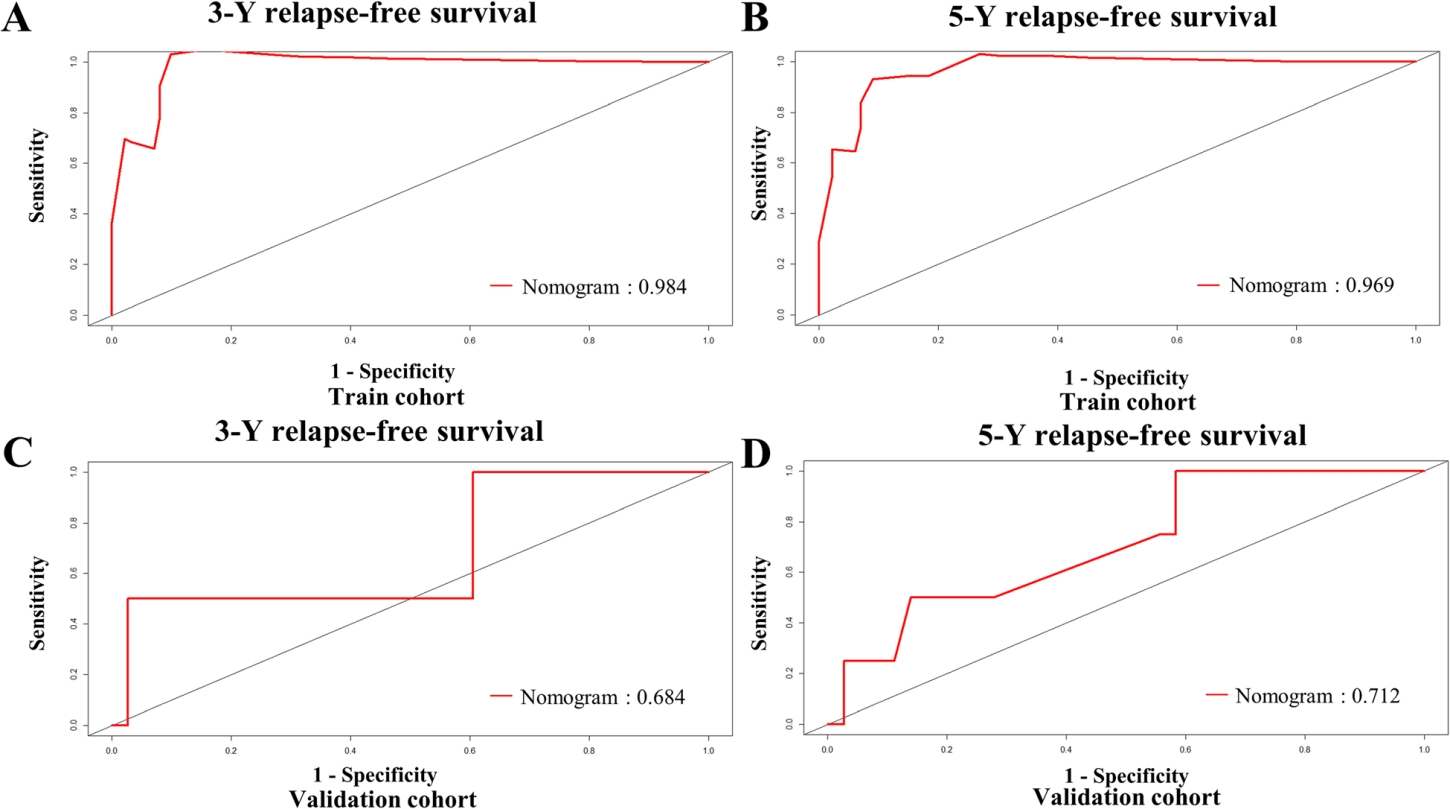
Receiver operating characteristic curve analysis for the sensitivity and specificity of the nomogram system to predict 3-y relapse-free survival (A, **C**) and 5-y relapse-free survival (B, **D**) in training and validation cohorts

To better verify the actual predictive power of the nomogram, the above results were verified using the verification group data, showing that the C index was 0.785 (95% CI: 0.614–0.957), and the five-year and three-year external calibration curves met those of the standard baseline (Fig. 3C and D). We also used the ROC curve to verify the nomogram performance (Fig. 4). The AUC values of the training and validation groups at 3 and 5 years were both greater than 0.65 and by comparing the AUC values of the two groups, the nomogram model were showing well accuracy of the nomogram in predicting RFS.

### Decision curve analysis

**Fig. 5:**
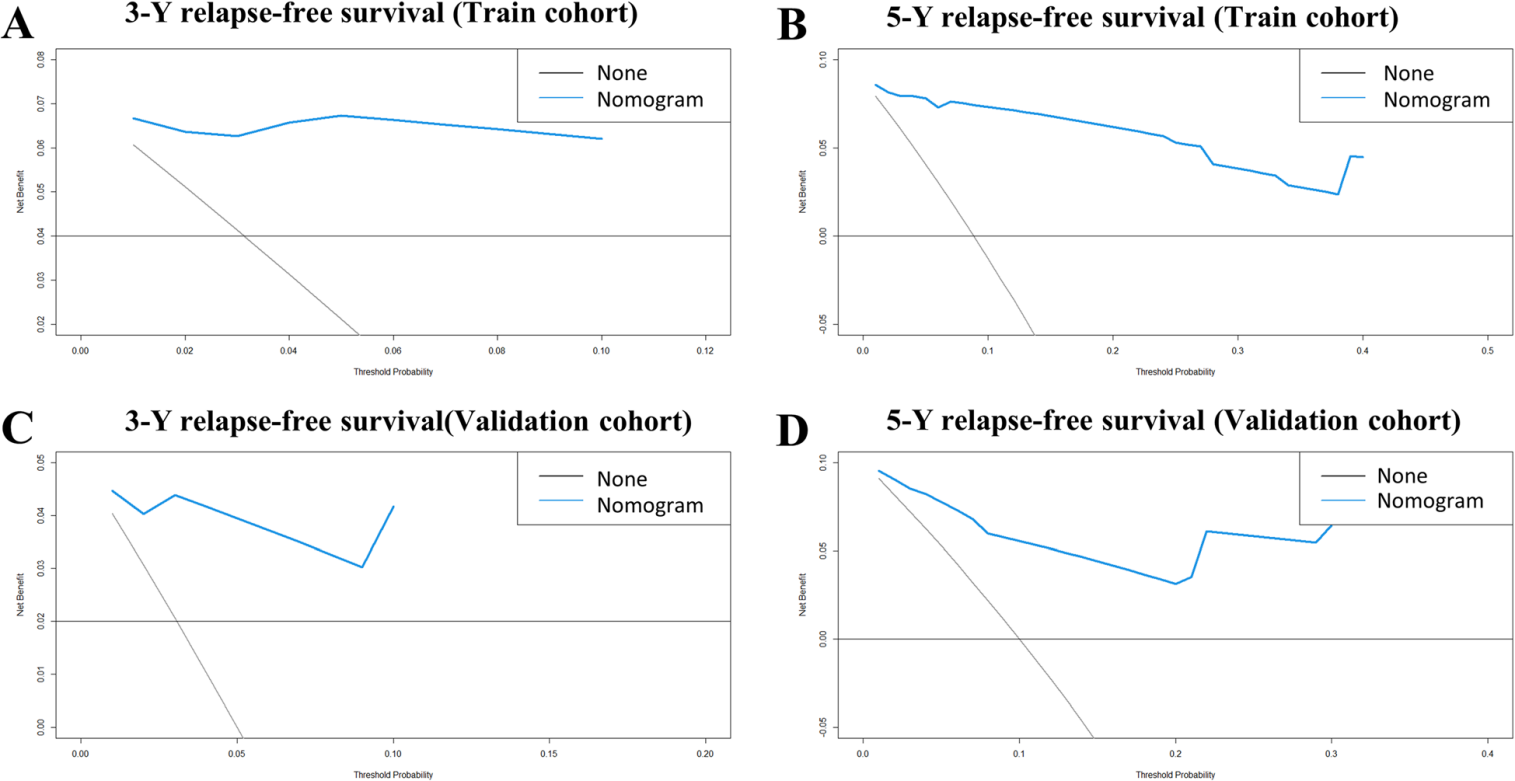
Decision curve analysis of the training cohort (**A**, **B**) and validation cohort (**C**, **D**) for 3- and 5- years relapse-free survival

Decision Curve Analysis (DCA) is a novel method for evaluating prognostic strategies that can evaluate the predictive power of prognostic models. Figure 5 shows the DCA curve of nomogram in the training and validation cohorts. The DCA of the nomogram has a high net benefit, which indicates that the nomogram can have well clinical application.

### Risk stratification of OS

**Fig. 6:**
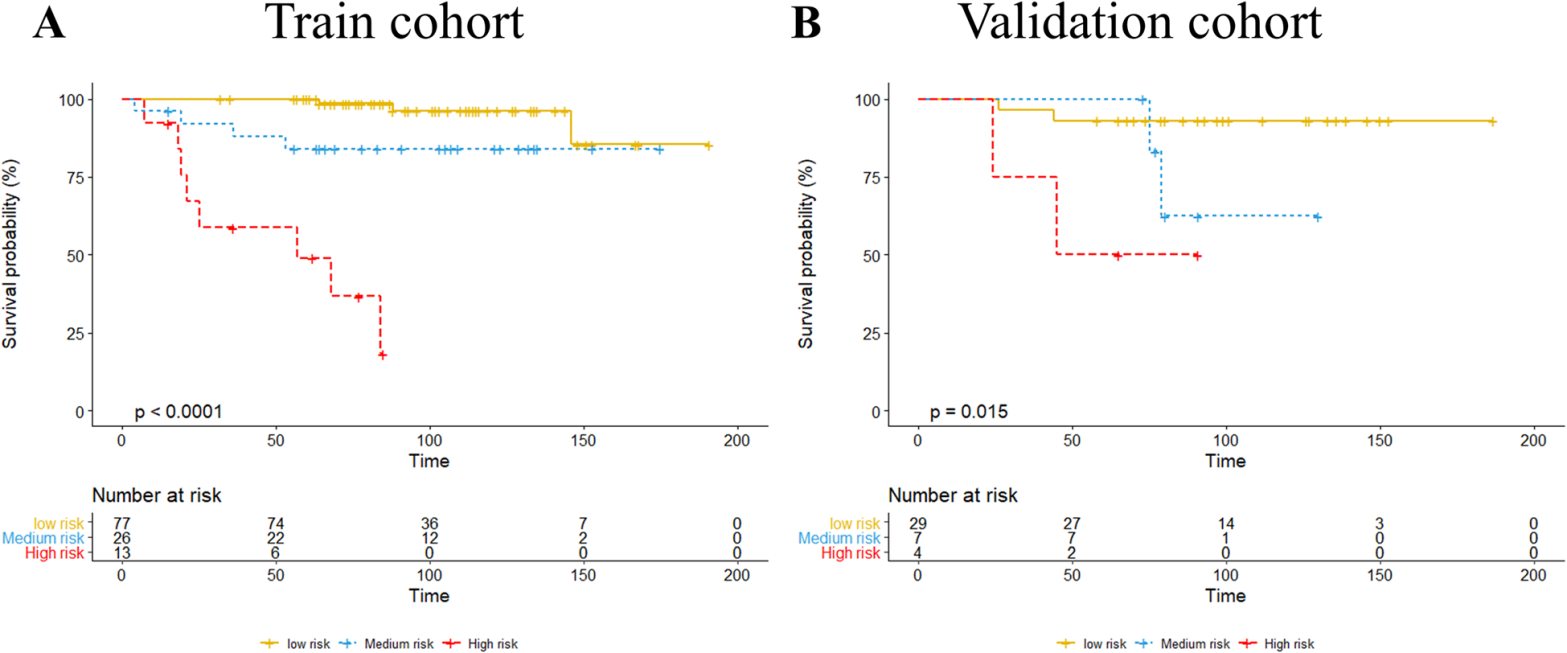
The relapse-free survival analysis of patients after risk-stratification (**A** for training cohort; **B** for validation cohort)

Based on the nomogram scores, patients were divided into low-risk (0–100 points), medium-risk (101–179 points), and high-risk (180 points or higher) subgroups. In the training cohort, there were 77 patients in the low-risk group, 26 patients in the intermediate-risk group, and 13 patients in the high-risk group. In the validation cohort, 29 patients were included in the low-risk group, 7 patients were included in the medium-risk group, and 4 patients were included in the high-risk group. There were significant differences in the incidence of RFS among the subgroups, and the relapse-free survival rate of the high-risk subgroup was lower than that of the other groups (*P* < 0.05) (Fig. 6).

## Discussion

In this study, we obtained results from 156 patients at SYSUCC, and ALB, NLR, T stage, and WHO histologic types were found to be independent prognostic factors of RFS. We developed a nomogram that can effectively predict the RFS rate of patients with thymic epithelial tumors at 3 and 5 years.

Slow disease progression and good prognosis have largely limited research on thymic epithelial tumors. At the same time, in the era of precision medicine, it is very important to analyze patient information as comprehensively as possible to screen factors affecting the prognosis for treatment decision-making. At present, the Masaoka-Koga staging system is still the gold standard for predicting the prognosis of thymic epithelial tumors. However, similar to the results of Fukui et al., [7] our results show that the T staging is better than the Masaoka-Koga staging in predicting prognosis. Therefore, the nomogram developed in this study was mainly included T stage. Based on this, the nomogram along with the patient’s various clinical and hematological indicators was used to construct a prognostic model. The nomogram has the advantage of a multi-dimensional comprehensive prediction of prognosis [24]. Some studies have reported nomograms related to the prognosis of thymic epithelial tumors. However, they considered only the clinicopathological characteristics or treatment methods of patients and do not consider relevant hematological indicators. At the same time, many studies on hematological indicators have shown that Hb, NE, LY, platelet (PLT), NLR, ALB, GLB, ALB/GLB, platelet-lymphocyte ratio (PLR), NLR, and SII [12, 13, 15, 25] have potential to become prognostic hematological indicators for various tumors including thymic epithelial tumors.

Our nomogram is composed of several factors that affect prognosis, which are commonly used in clinical practice. The nomogram showed that higher WHO pathological stage was related to poor RFS in patients with thymic epithelial tumors. This finding is consistent with the findings of other studies [26]. In terms of T staging, lower T staging can result in a more satisfactory RFS. In the studies of other researchers, the patient’s preoperative clinical staging was a factor affecting the prognosis of thymic epithelial tumors [27]. At the same time, we found that elevated NLR was associated with poor prognosis in patients with thymic epithelial tumors. This is the same as other people’s research results [6, 28]. In addition, a higher preoperative serum ALB level can often result in a more satisfactory prognosis, which is consistent with the findings of other studies reporting better prognosis in patients with thymic epithelial tumors with higher ALB levels [15].

In predicting the prognosis of certain cancers, nomograms have been developed and proven to be more accurate than traditional staging systems [29, 30]. Therefore because thymectomy is an effective treatment for thymic epithelial tumors, [26, 31–33] we constructed a prognostic nomogram for patients with thymic epithelial tumors after surgery. The nomogram performs well in predicting the survival rate. Its prediction is supported by the C index (0.902 and 0.785 for the training and verification cohorts, respectively), and the calibration curve was consistent with that of the baseline. The nomogram has high accuracy in predicting survival, and the DCA curve also showed that the nomogram had well predictive ability.

To the best of our knowledge, this is a relatively new attempt to develop a prognostic RFS nomogram for patients with thymic epithelial tumors by combining hematological and clinical indicators. Although this nomogram did not include many hematological indicators in the end, it combines clinical indicators, hematological indicators and other important clinical information to achieve the ultimate goal of integrating multi-dimensional data to jointly predict the prognosis of thymic epithelial tumors.

This study has several limitations. First, this was a retrospective study. Moreover, this was only a single-center study that included a small number of patients. Further research including more number of cases is still needed to verify our results. Second, tumor markers (CEA, SCC, AFP, etc.) and other potentially valuable hematological indicators were not included in this study. Third, the dynamic changes in hematological indicators considered in this study were not followed up after the operation.

## Conclusions

In summary, by combining hematological and clinical indicators, we established and validated a nomogram for predicting the relapse-free survival of patients with thymic epithelial tumors. This convenient nomogram had well performance to distinguish the prognosis and risk of patients. Our findings suggest that it may be a potentially easy-to-use tool for physicians and can aid in postoperative personalized prognosis assessment and early identification of high-risk patients. Although the nomogram appears useful for prognostication and identifying high-risk patients, further prospective studies are needed to validate the nomogram and confirm the contribution of each prognostic factor.

## Supplementary Information

**Additional file 1.** KM analysis of ALB based on relapse-free survival.

**Additional file 2.** KM analysis of T stage based on relapse-free survival.

**Additional file 3.** KM analysis of Invasion of great vessels based on relapse-free survival.

**Additional file 4.** KM analysis of tumor capsule status based on relapse-free survival.

**Additional file 5.** KM analysis of SII based on relapse-free survival.

**Additional file 6.** KM analysis of WHO Histology based on relapse-free survival.

**Additional file 7.** KM analysis of NE based on relapse-free survival.

**Additional file 8.** KM analysis of PLR based on relapse-free survival.

**Additional file 9.** KM analysis of NLR based on relapse-free survival.

## Data Availability

Data from this study are available to any interested researchers upon reasonable request to the corresponding author.

## Abbreviations

NLR: Neutrophil-to-lymphocyte ratio
Hb: Hemoglobin
ALB: Albumin
BMI: Body mass index
NE: Neutrophil count
LY: Lymphocyte count
GLB: Globulin
SII: Systemic immune-inflammation Index
PLT: Platelet
PLR: Platelet-lymphocyte ratio
pT stage: Pathological T stage
OS: Overall survival
C-index: Consistency index
ROC: Receiver operating characteristic
DCA: Decision curve analysis
KM: Kaplan-Meier
RFS: Relapse-free survival.
