# Supplementary figures and images for: Nomogram predict relapse-free survival of patients with thymic epithelial tumors after surgery
Source: BMC Cancer. 2021 Jul 22;21:847. doi: 10.1186/s12885-021-08585-y (PMC8299634; doi:10.1186/s12885-021-08585-y)

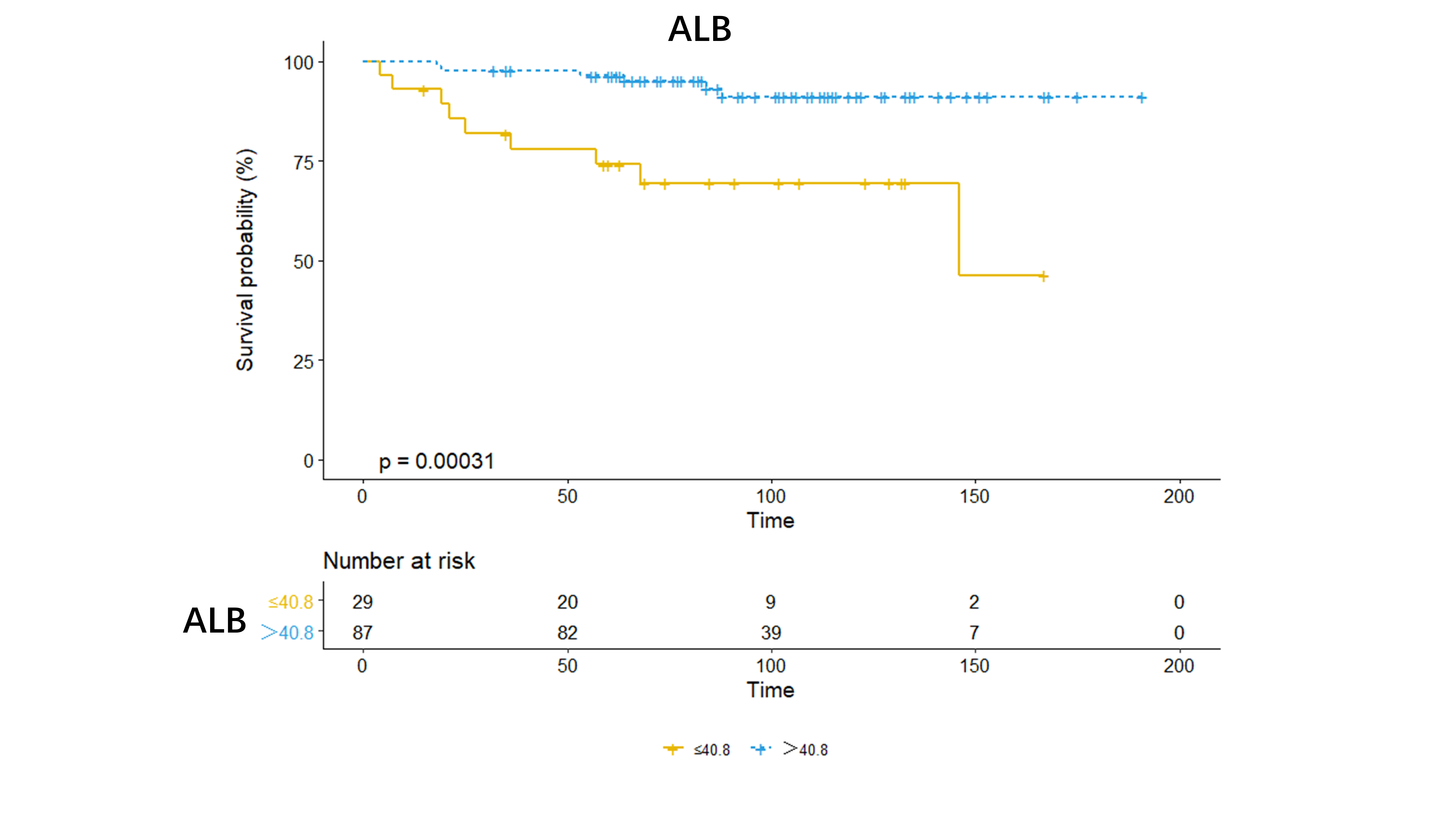

Supplement: Supplementary file 1 — Additional file 1. KM analysis of ALB based on relapse-free survival. [file 12885_2021_8585_MOESM1_ESM.tif]

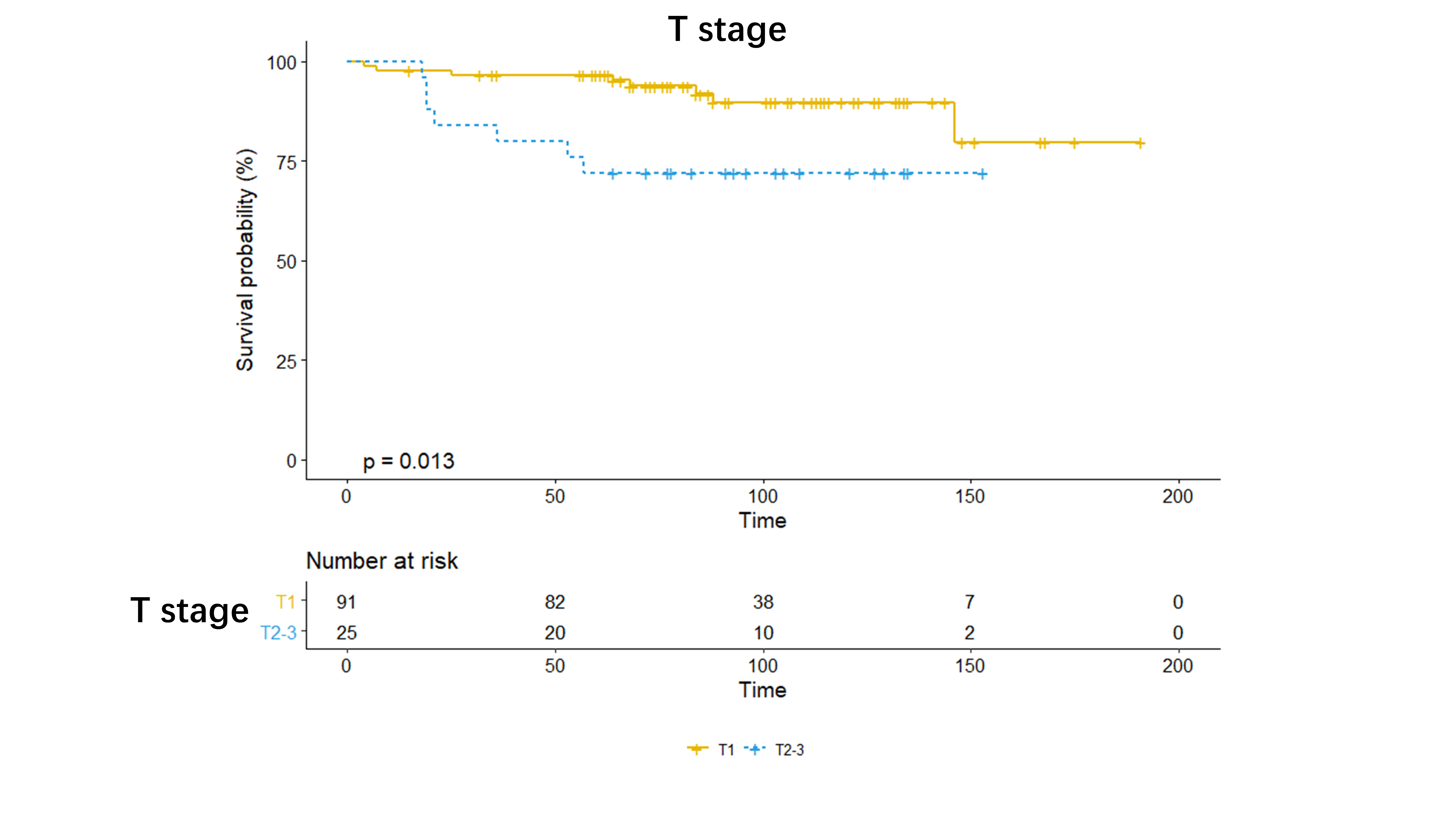

Supplement: Supplementary file 2 — Additional file 2. KM analysis of T stage based on relapse-free survival. [file 12885_2021_8585_MOESM2_ESM.tif]

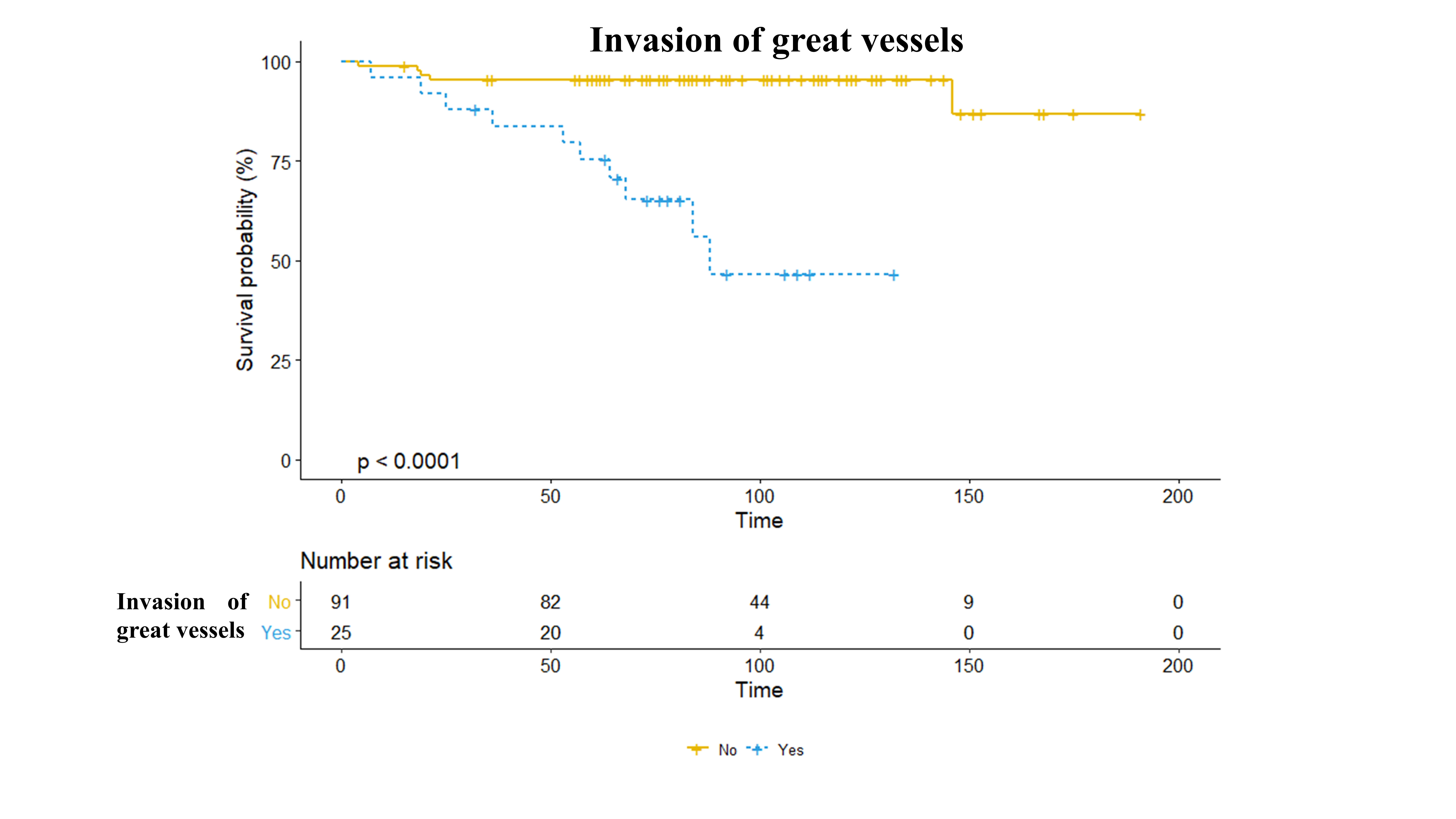

Supplement: Supplementary file 3 — Additional file 3. KM analysis of Invasion of great vessels based on relapse-free survival. [file 12885_2021_8585_MOESM3_ESM.tif]

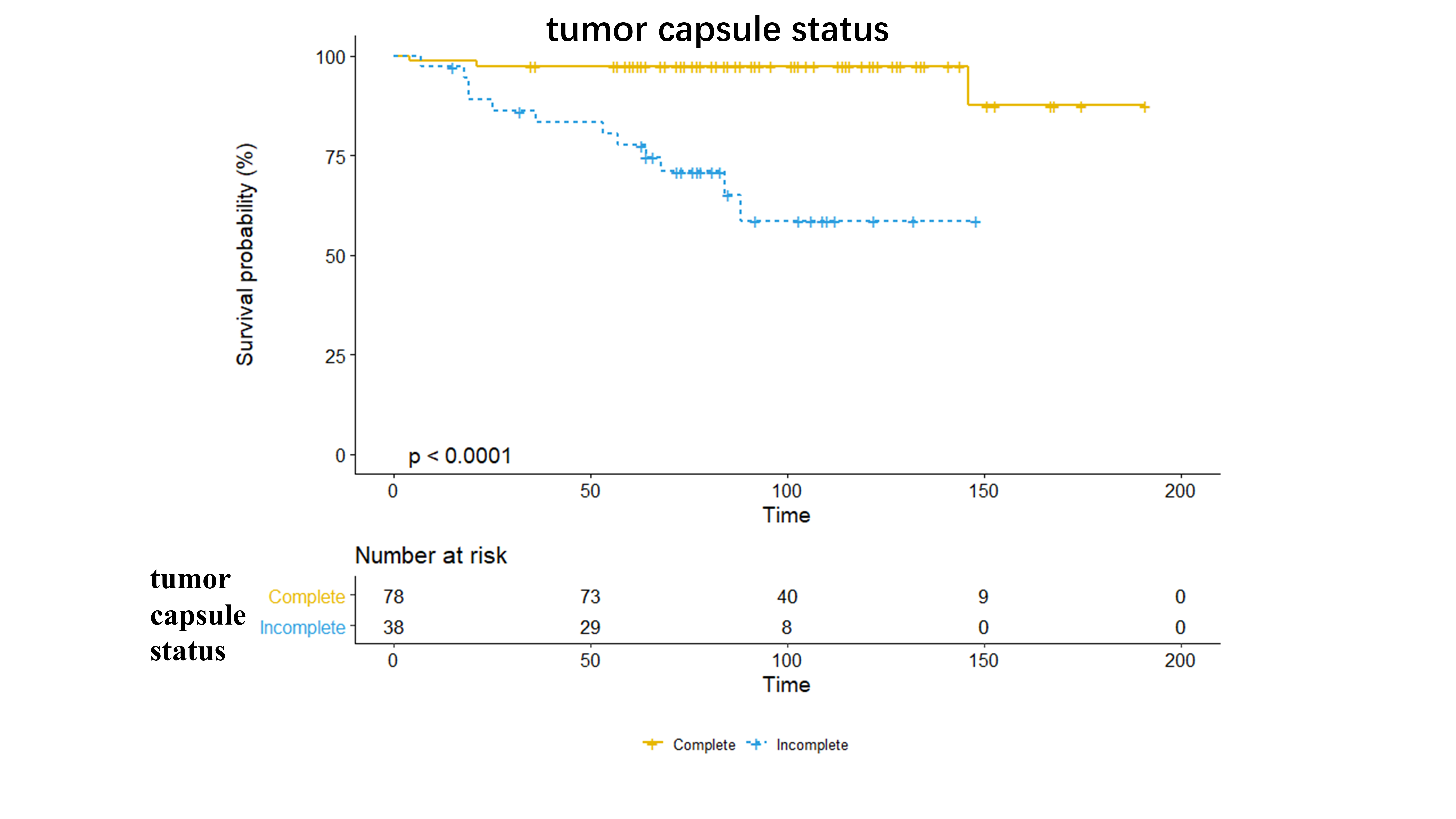

Supplement: Supplementary file 4 — Additional file 4. KM analysis of tumor capsule status based on relapse-free survival. [file 12885_2021_8585_MOESM4_ESM.tif]

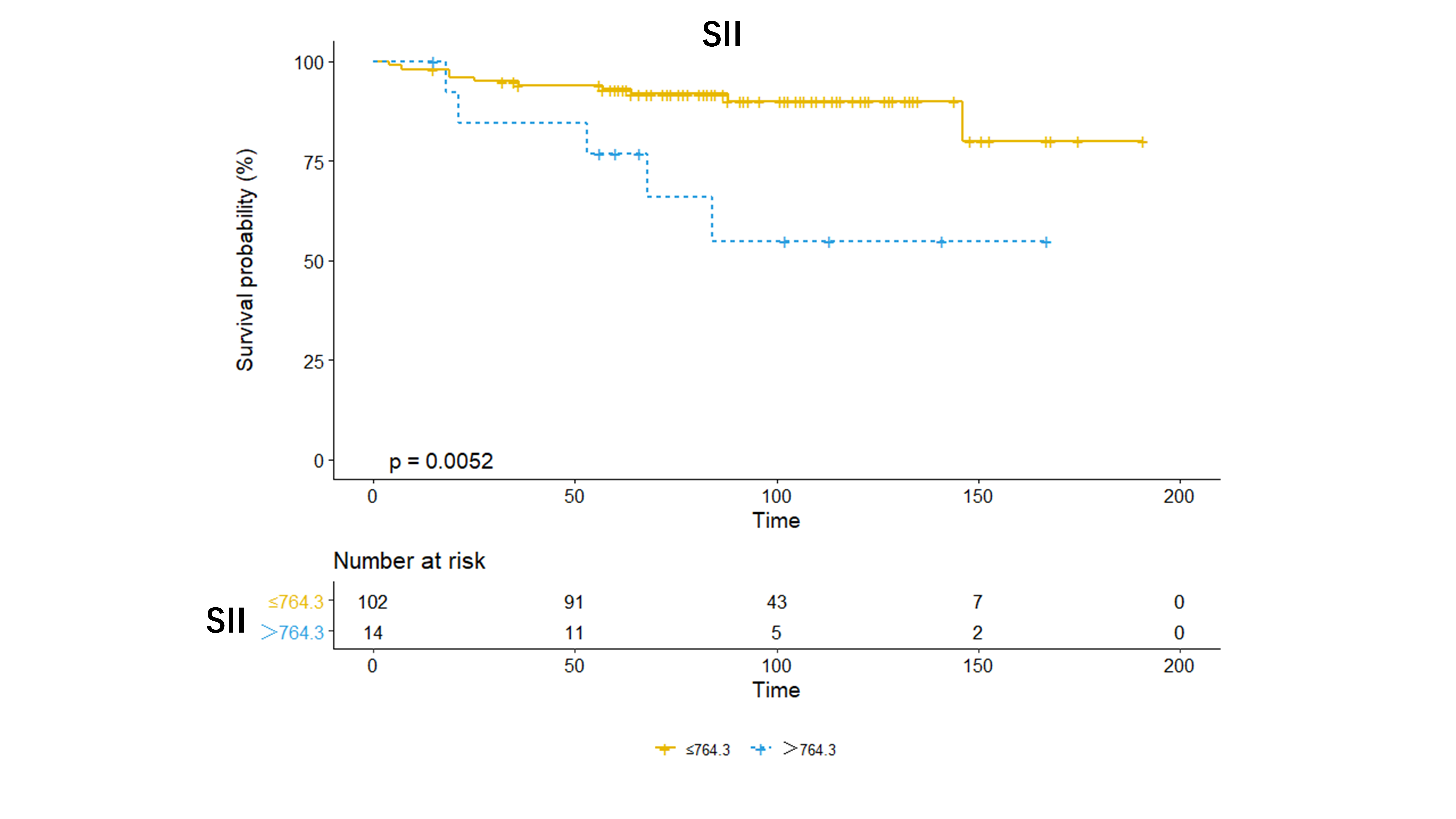

Supplement: Supplementary file 5 — Additional file 5. KM analysis of SII based on relapse-free survival. [file 12885_2021_8585_MOESM5_ESM.tif]

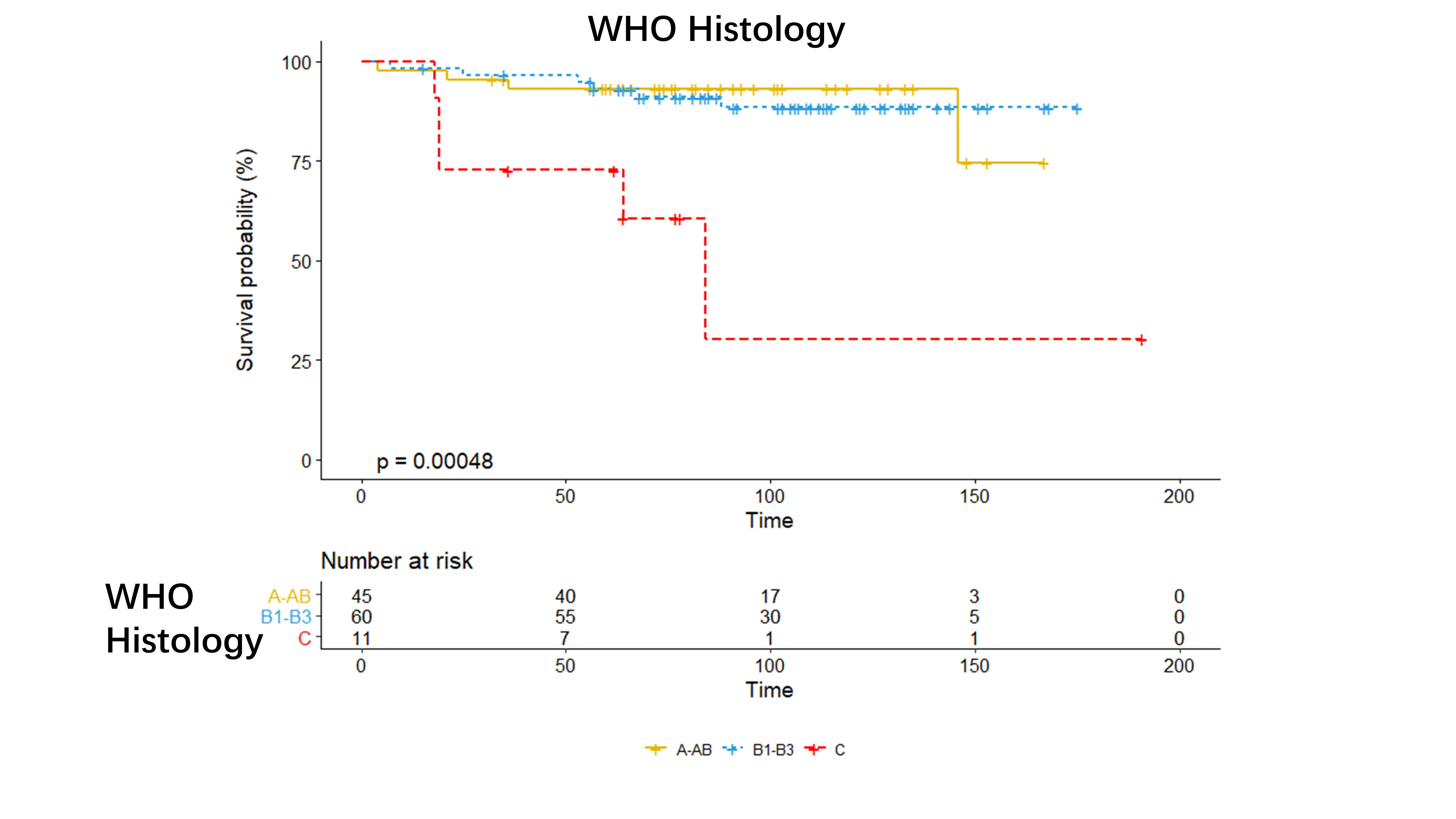

Supplement: Supplementary file 6 — Additional file 6. KM analysis of WHO Histology based on relapse-free survival. [file 12885_2021_8585_MOESM6_ESM.tif]

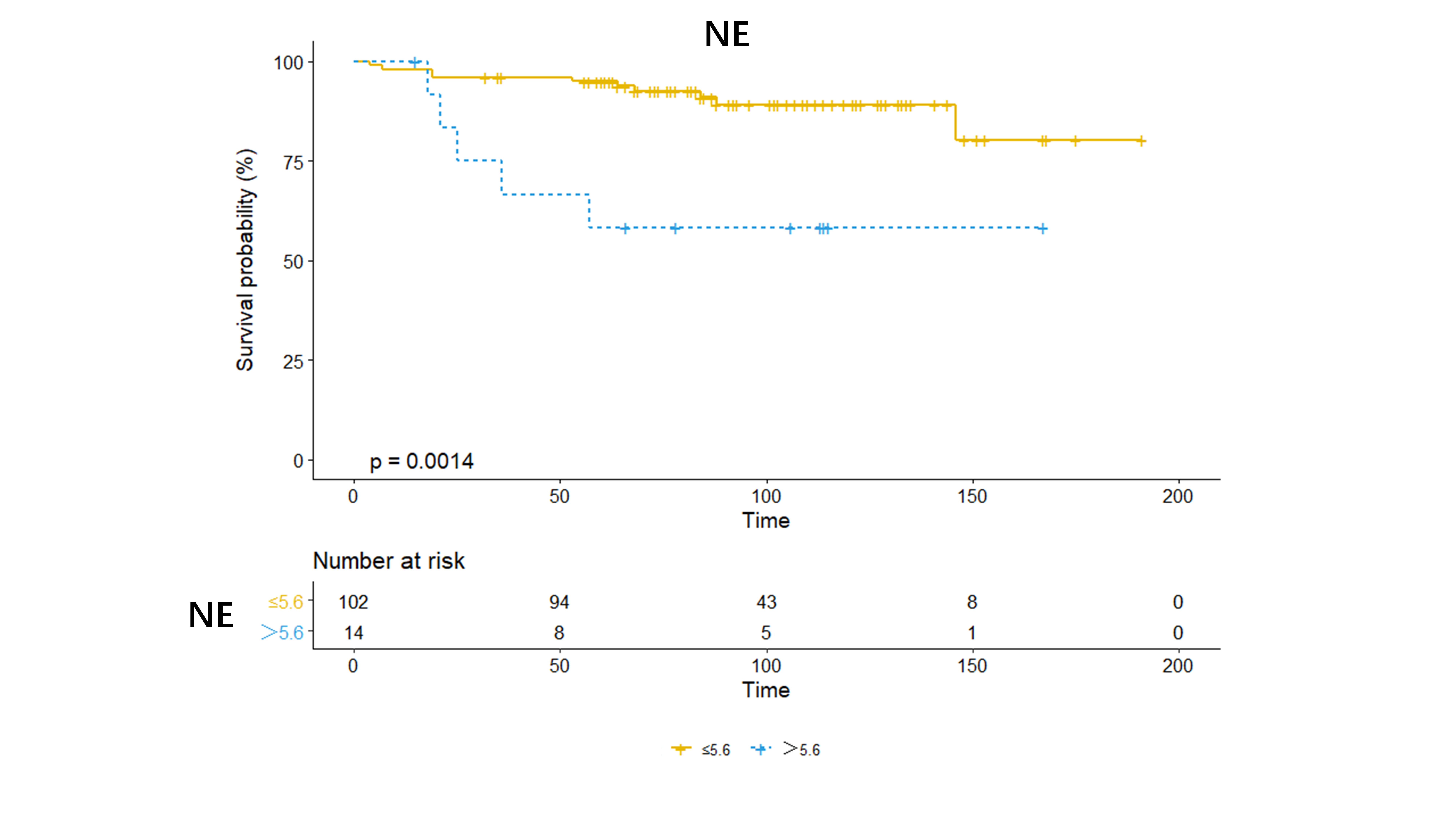

Supplement: Supplementary file 7 — Additional file 7. KM analysis of NE based on relapse-free survival. [file 12885_2021_8585_MOESM7_ESM.tif]

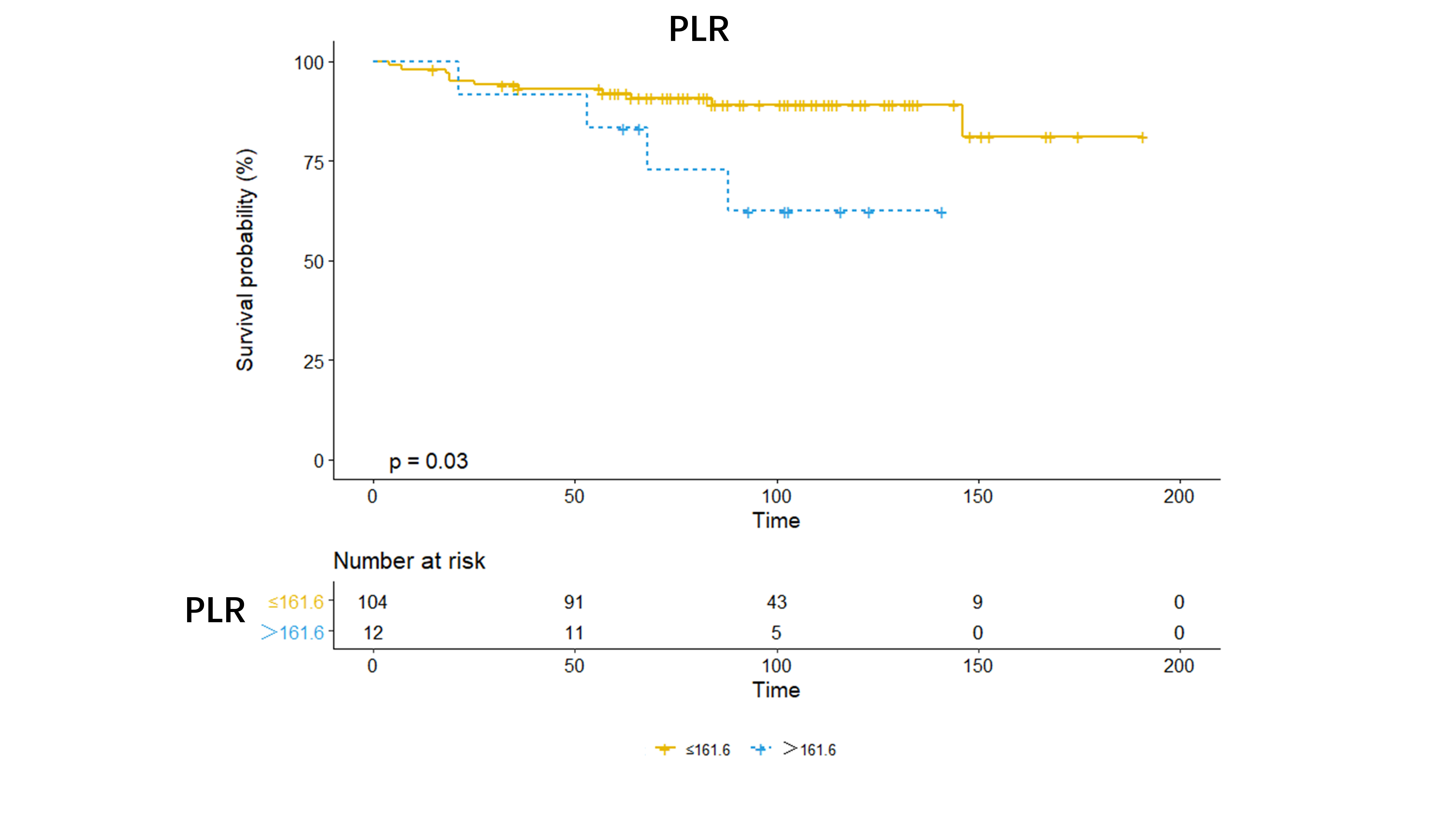

Supplement: Supplementary file 8 — Additional file 8. KM analysis of PLR based on relapse-free survival. [file 12885_2021_8585_MOESM8_ESM.tif]

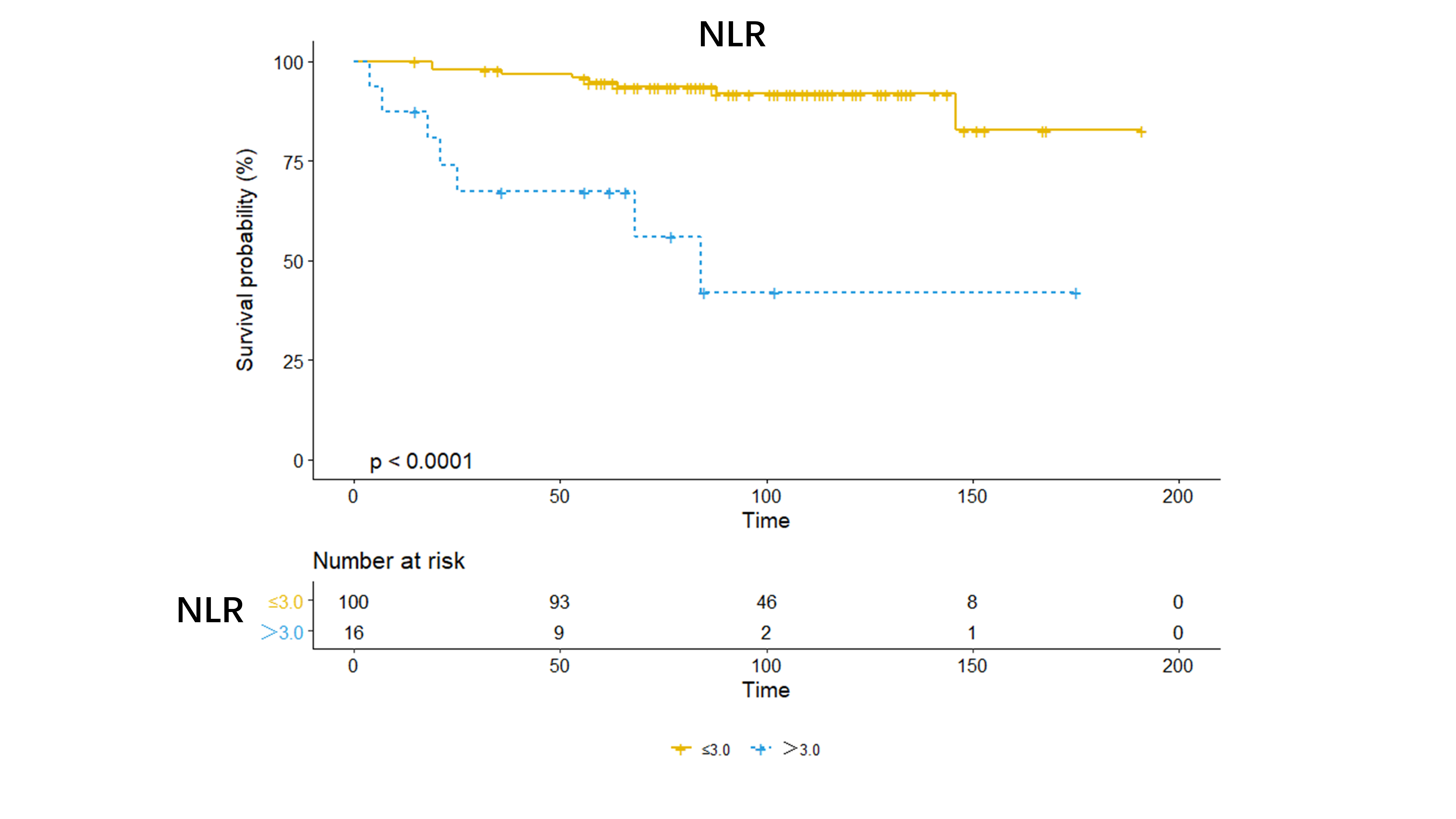

Supplement: Supplementary file 9 — Additional file 9. KM analysis of NLR based on relapse-free survival. [file 12885_2021_8585_MOESM9_ESM.tif]
